# The Effect of Cucurbit[7]uril on the Antitumor and Immunomodulating Properties of Oxaliplatin and Carboplatin

**DOI:** 10.3390/ijms22147337

**Published:** 2021-07-08

**Authors:** Ekaterina Pashkina, Alina Aktanova, Irina Mirzaeva, Ekaterina Kovalenko, Irina Andrienko, Nadezhda Knauer, Natalya Pronkina, Vladimir Kozlov

**Affiliations:** 1 Research Institute of Fundamental and Clinical Immunology, 14 Yadrintsevskaya St, 630099 Novosibirsk, Russia; aktanova_al@mail.ru (A.A.); nuknauer@niikim.ru (N.K.); pronkina@yandex.ru (N.P.); vakoz40@yandex.ru (V.K.); 2 Department of Clinical Immunology, Novosibirsk State Medical University, 52 Krasny Prospect, 630091 Novosibirsk, Russia; 3 Nikolaev Institute of Inorganic Chemistry, Siberian Branch of the Russian Academy of Sciences, 3 Lavrentiev Ave, 630090 Novosibirsk, Russia; dairdre@gmail.com (I.M.); e.a.kovalenko@niic.nsc.ru (E.K.); andrienko@niic.nsc.ru (I.A.)

**Keywords:** cucurbit[7]uril, carboplatin, oxaliplatin, supramolecular complex, cell viability

## Abstract

Cucurbit[7]uril (CB[7]) is a molecular container that may form host–guest complexes with platinum(II) anticancer drugs and modulate their efficacy and safety. In this paper, we report our studies of the effect of CB[7]–oxaliplatin complex and the mixture of CB[7] and carboplatin (1:1) on viability and proliferation of a primary cell culture (peripheral blood mononuclear cells), two tumor cell lines (B16 and K562) and their activity in the animal model of melanoma. At the same time, we studied the impact of platinum (II) drugs with CB[7] on T cells and B cells in vitro. Although the stable CB[7]–carboplatin complex was not formed, the presence of cucurbit[7]uril affected the biological properties of carboplatin. In vivo, CB[7] increased the antitumor effect of carboplatin, but, at the same time, increased its acute toxicity. Compared to free oxaliplatin, its complex with CB[7] shows a greater cytotoxic effect on tumor cell lines B16 and K562, while in vivo, the effects of the free drug and encapsulated drug were comparable. However, in vivo studies also demonstrated that the encapsulation of oxaliplatin in CB[7] lowered the toxicity of the drug.

## 1. Introduction

**Figure 1:**
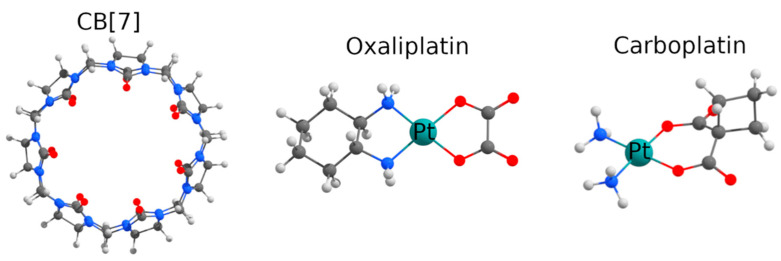
Molecular structures of CB[7], oxaliplatin and carboplatin.

Cucurbit[n]urils (CB[n]s) are barrel-shaped macrocycles that may form host–guest complexes with drugs and, thus, may be used as nanosized vehicles for drug delivery. To date, CB[n]s have been reported to form complexes with a variety of pharmaceuticals, including prilocain [1], isoniazid [2], sanguinarine [3], berberine [4], pilocarpine [5], tuftsin [6], mitoxantrone [7], methotrexate [8] and other peptides or proteins [9]. The effect of complexation with CB[n] on the biological properties of encapsulated drugs was also studied and discussed [10]. A particular research interest is focused on complexes of CB[n] with anticancer drugs based on metal complexes, primarily platinum (II). It is known that CB[7] forms complexes with various platinum compounds, and complexation can enhance the antitumor effect of the drug and reduce side effects [11].The most studied system of this type is the complex of CB[7] (Figure 1) with cisplatin [12]. In vivo studies have shown that the complexation with CB[n] reduces the toxic side effects of cisplatin [13,14]. In most cases, complexation with CB[7] does not lead to a decrease in the cytotoxic activity of cisplatin. Moreover, the complexation with CB[7] overcomes the specific cisplatin resistance in the cisplatin-resistant A2780/cp70 cell line [15,16,17]. Plumb et al. show that the cisplatin–CB[7] complex was as effective against a cisplatin-resistant tumor formed by A2780 cells as free cisplatin [15]. In a case of cisplatin-resistant A2780/CP70 xenografts, the complex reduced tumor growth 1.6-fold compared to free cisplatin. The ability of the cisplatin–CB[7] complex to overcome resistance in vivo apparently results from the pharmacokinetic effect.

The effect of encapsulation in CB[n] on the chemical and biological properties of other widely used platinum drugs, such as oxaliplatin and carboplatin (Figure 1), are yet insufficiently studied. The encapsulation of oxaliplatin in CB[7] (Figure S2 in Supplementary) greatly increased the stability of the complex and reduced some side effects, but also reduced the antitumor activity of the drug (A549 human non-small cell lung, SKOV-3 human ovarian, SKMEL-2 human melanoma, XF-498 human CNS and HCT-15 human colon cell lines were tested [18]). However, more recent research on HCT116 and HT29 colorectal tumor cell lines [19] showed that the CB[7]–oxaliplatin complex demonstrated an enhancement of antitumor activity compared to free oxaliplatin and free CB[7], which appeared to have its own slight antitumor activity towards these cell lines, associated with CB[7]’s ability to bind spermine, which is essential for tumor growth. Therefore, it is important to test the antitumor activity of the CB[7]–oxaliplatin complex towards other cancer cell lines, to search for similar effects.

The inclusion complex of CB[7] with carboplatin has not yet been obtained. DFT calculations showed that such a complex could hypothetically exist, but the series of CB[n]–carboplatin complexes would be the least stable among the systems considered in that study [20]. DFT calculations show that the formation of such a complex is less energetically advantageous compared to CB[7] complexes with other Pt complexes. Moreover, experimentally, the carboplatin–CB[7] complex has not yet been obtained, despite some recurring research efforts. However, interaction of CB[7] with carboplatin provokes aquation of the latter, and one of the products of this process forms a complex with CB[7]. As we found earlier, CB[7] induces the aquation of carboplatin with the formation of CB[7] inclusion complex with one of the aquation products [21,22,23]. This process differs in PBS buffer solution and RPMI-1640 medium, because CB[7] could bind to some components of the media, such as amino acids or proteins. However, in all cases, CB[7] had a certain impact on the carboplatin aquation rate. Thus, we may expect CB[7] to change the biological properties of carboplatin, even without the formation of a stable CB[7]–carboplatin inclusion complex.

The biological effect of platinum(II) anticancer compounds is not limited to their ability to inhibit cell proliferation; they also have an immunomodulatory effect [24]. For instance, cisplatin significantly inhibited the production of IL-2 by PBMCs in vitro [25]. However, effects of other platinum(II) drugs and, moreover, their complexes with CB[7] on immune cells are not entirely clear. In this study, we investigated the effect of platinum compounds with CB[7] on the primary culture of immunocompetent cells, PBMCs.

In this paper, we report our studies of the cytotoxic and cytostatic effects of the CB[7]–oxaliplatin complex and the 1:1 mixture of carboplatin with CB[7] (we selected this ratio because the complex of carboplatin aquation product with CB[7] was formed in a 1:1 ratio [21]) on the primary cell culture (mononuclear cells of the peripheral blood) and tumor cell lines B16 and K562, as well as in vivo studies of the CB[7]–oxaliplatin complex and the 1:1 CB[7] with carboplatin mixture antitumor activity and acute toxicity.

## 2. Results and Discussion

### 2.1. In Vitro Cytotoxicity and Immunomodulating Properties

**Figure 2:**
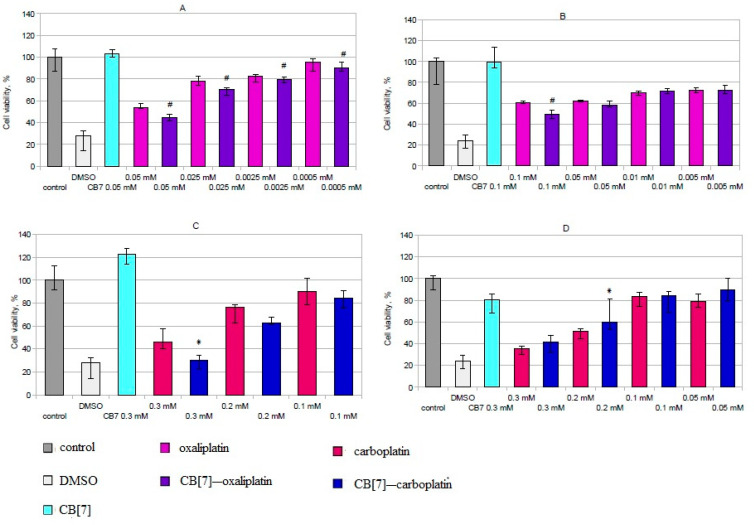
Effects of the CB[7] and platinum drugs on cell viability of different cell line. (**A**) Effects of the oxaliplatin–CB[7] complex on B16 cells’ viability. (**B**) Effects of the oxaliplatin–CB[7] complex on K562 cells’ viability. (**C**) Effects of the mixture of CB7 and carboplatin (1:1) on viability of B16 cells. (**D**) Effects of the mixture of CB7 and carboplatin (1:1) on viability of K562 cells. Data are expressed as the median with interquartile range. * Indicates a significant difference (*p* < 0.05) vs. carboplatin. # Indicates a significant difference (*p* < 0.05) vs. oxaliplatin.

**Figure 3:**
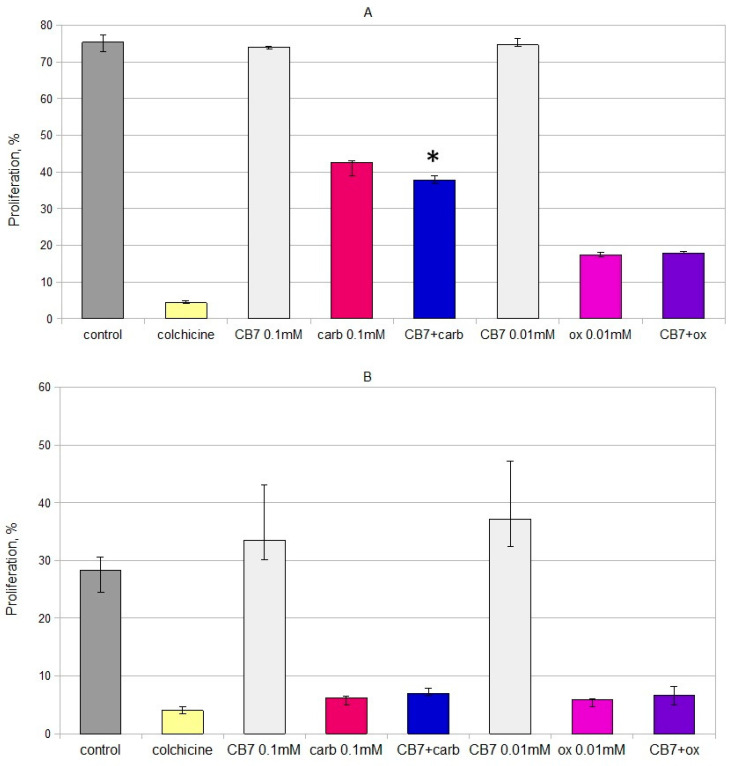
Effects of the CB[7] and platinum drugs on proliferation activity of different cell line. (**A**) Effects of the oxaliplatin–CB[7] complex and the mixture of CB7 and carboplatin (1:1) on proliferation of B16 cells. * Indicates a significant difference (*p* < 0.05) vs. carboplatin. (**B**) Effects of the oxaliplatin–CB[7] complex and the mixture of CB7 and carboplatin (1:1) on proliferation of K562 cells. Abbreviations: carb—carboplatin; ox—oxaliplatin. Data are expressed as the median with interquartile range.

Oxaliplatin and the CB[7]–oxaliplatin complex exerted a cytotoxic effect on B16 cells at a concentration of 2.5 µM and above (Figure 2A). At all concentrations, the CB[7]–oxaliplatin complex reduced the viability of B16 myeloma cells more than free oxaliplatin. Both oxaliplatin and the CB[7]–oxaliplatin complex suppressed the K562 cells’ viability at concentrations above 0.001 mM (Figure 2B). At the same time, the CB[7]–oxaliplatin complex significantly reduced the viability of K562 cells in comparison with free oxaliplatin in high concentration (0.1 mM). The complexation of oxaliplatin with CB[7] did not lead to a higher suppression of proliferation (Figure 3).

The mixture of CB[7] and carboplatin (1:1) had an enhanced cytotoxic effect on cultures of murine B16 melanoma in comparison with carboplatin. Thus, carboplatin led to a decrease in the viability of B16 culture cells at concentrations of 0.2 and 0.3 mM, while adding CB[7] with carboplatin in a 1:1 ratio caused a decrease in viability at concentrations of 0.1, 0.2 and 0.3 mM. When cells were cultivated with CB[7] and carboplatin (1:1) at a concentration of 0.3 mM, the viability of B16 cells was significantly lower than in the presence of carboplatin at the same concentration (Figure 2C). Additionally, the addition of CB[7] enhances the cytostatic effect of carboplatin on cells of murine B16 melanoma (Figure 2A).

The presence of carboplatin or its mixture with CB[7] (1:1) suppresses cell viability of K562 cells at concentrations of 0.1, 0.2 and 0.3 mM (Figure 2D). Significant differences between the effect of carboplatin and carboplatin with CB[7] at concentrations of 0.1 and 0.3 mM were not observed. However, at a concentration of 0.2 mM, carboplatin with CB[7] had a lower effect on cell viability than carboplatin only. Carboplatin in mixture with CB[7] (1:1) inhibited cell proliferation of the K562 line; there were no significant differences between the effect of carboplatin with CB[7] in a 1:1 ratio and free carboplatin (Figure 3B). Different effects on B16 and K562 cells also confirmed our hypothesis that CB[7] does not enhance the effect of carboplatin on cells that derived from hematopoietic stem cells. It is known that encapsulation of oxaliplatin in CB[7] can affect the antitumor effect [17,18], which may depend on the cell lines.

**Figure 4:**
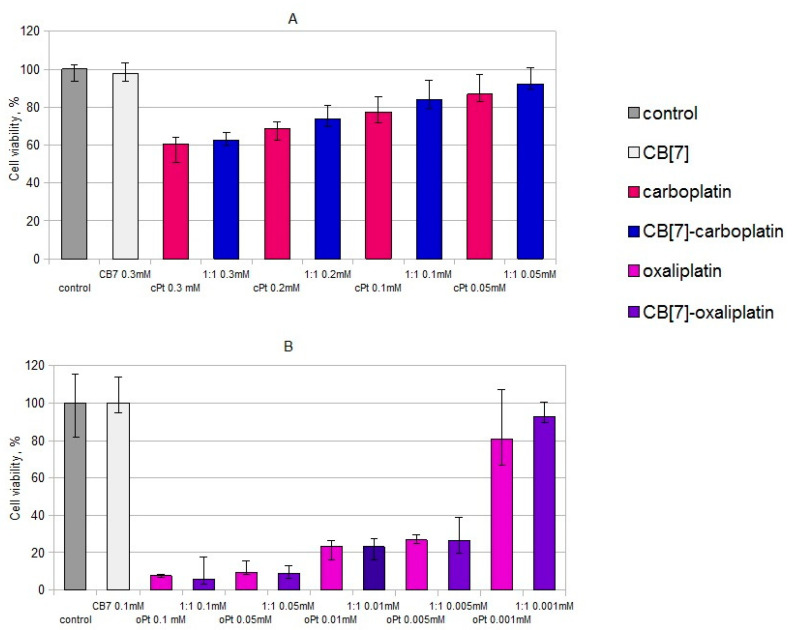
Effects of the CB[7] and platinum drugs on cell viability of PBMCs from healthy donors. (**A**) Effects of the mixture of CB7 and carboplatin (1:1) on cell viability of PBMCs. (**B**) Effects of the oxaliplatin–CB[7] complex on PBMCs’ viability.

In the study of the cytotoxic effect of the oxaliplatin complex with CB[7] on the primary culture of PBMCs, it was shown that the complex and free oxaliplatin suppressed the viability of these cells at concentrations of 0.005 mM and above (Figure 4B). Significant differences between the effects of oxaliplatin and the complex of oxaliplatin with CB[7] were not found. Carboplatin and the CB7–carboplatin mixture (1:1) decreased viability of PBMCs at concentrations of 0.1, 0.2 and 0.3 mM (Figure 4A). Significant differences between the cytotoxic effect of carboplatin and carboplatin with CB[7] (1:1) on PBMCs were not observed. Thus, the complexation of platinum compounds with CB[7] did not enhance the cytotoxic effect on PBMCs. According to the literature, oxaliplatin has quite high cytotoxicity compared to normal colorectal cells, while oxaliplatin–CB[7] has significantly lower cytotoxicity [18]. Therefore, the cytotoxicity of oxaliplatin for non-cancer cells was not enhanced by encapsulating it with CB[7], thus improving the biosafety of oxaliplatin during the delivery process.

**Figure 5:**
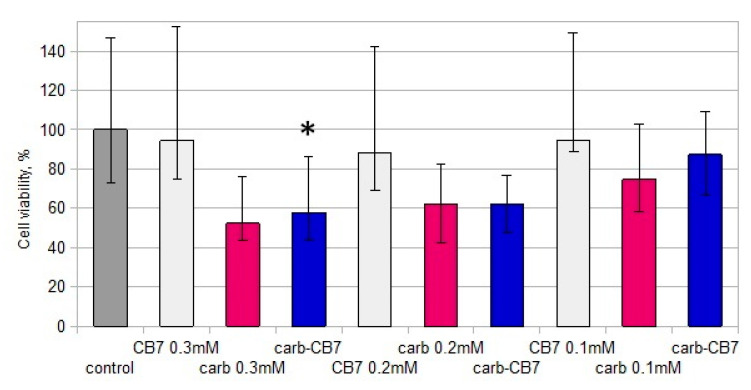
Effects of the CB[7] and platinum drugs on cell viability of PBMCs from patients with B-cell lymphomas.* Indicates a significant difference (*p* < 0.05) vs. carboplatin.

Based on the effect of carboplatin with CB[7] on PBMCs and K562, we suggested that adding CB[7] did not enhance the cytotoxic effect of carboplatin on cells that derived from hematopoietic stem cells. To confirm this hypothesis, we used PBMCs obtained from patients with B-cell lymphomas; most of the cells (60.3 ± 5.11% of PBMCs) in the primary culture were tumor cells (Figure 5). It was found that carboplatin with CB[7] (1:1) slightly reduced the viability of PBMCs from patients than carboplatin alone. Therefore, it seems that CB[7] reduces the toxic effect of carboplatin on healthy immune cells, as well as on tumor cells developed from immunocompetent cells; the mechanism of such action requires further study.

Oxaliplatin and oxaliplatin–CB[7] complex, as well as carboplatin and mixture of carboplatin with CB[7] (1: 1), did not affect the spontaneous proliferative activity of the PBMCs, but at the same time, they suppressed the anti-CD3-induced proliferative response, indicating an immunosuppressive effect on activated lymphocytes (Table 1 and Table 2).

Interestingly, the presence of carboplatin with CB[7] increased the relative number of CD3^+^CD4^+^ lymphocytes (T helpers) in both non-activated and activated cultures. At the same time, the number of cytotoxic T lymphocytes decreased proportionally in the activated culture. In addition, addition of oxaliplatin or oxaliplatin–CB[7] complex affected the subpopulation of PBMCs, causing an increase in the relative number of T-helper cells and reducing the number of killer T cells, both without activation and with activation by anti-CD3 antibodies. No significant differences were found in T cells after treatment of PBMCs with oxaliplatin and the oxaliplatin–CB[7]complex. These results indicate that CB[7] does not affect the immunosuppressive properties of platinum drugs.

### 2.2. In Vivo Antitumor Activity and Side-Effect Evaluation

**Figure 6:**
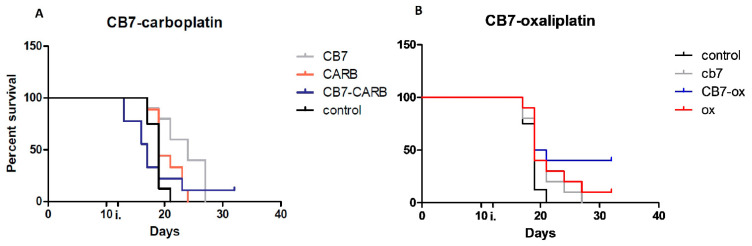
Effects of the CB[7] and platinum drugs on mice survival in melanoma model. (**A**) Impact of the mixture of CB7 and carboplatin (1:1) on mice survival. (**B**) Impact of the oxaliplatin–CB[7] complex on mice survival.

**Figure 7:**
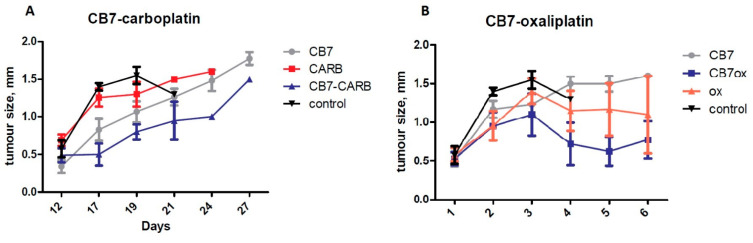
Effects of the CB[7] and platinum drugs on tumor size in melanoma mice model. (**A**) Impact of the mixture of CB7 and carboplatin (1:1) on tumor size. (**B**) Impact of the oxaliplatin–CB[7] complex on tumor size.

No statistically significant differences were observed between the survival rates of the groups that were treated by platinum drugs with or without CB[7] (Figure 6). It should be noted that in the first days after the administration of carboplatin with CB[7], the death rate of mice was associated not with the size of the tumor, but with the acute toxic effects, and therefore the survival curve does not reflect only the antitumor properties of these substances. Moreover, a similar toxic effect was not observed in groups of mice that received injections of only carboplatin or CB[7], which means that this effect can be explained by the synergistic effect of the components. Perhaps the synergistic effect is caused by higher toxicity of CB[7] complexes with products of the aquation of carboplatin, which are formed in vivo. During aquation, carboplatin decomposes into 1,1-cyclobutanedicarboxylic acid and cis-PtL_2_(NH_3_)_2_ (L = H_2_O or OH^−^), so it loses the leaving group and forms an inclusion complex with CB[7] [23]. Loss of the leaving group can increase the reactivity rate of products of the aquation of carboplatin with nucleophiles. Additionally, CB[7] significantly increased the survival rate compared to the control group treated with PBS only. At the same time, there was a significant decrease in tumor size in the group after administration of carboplatin with CB[7] compared to mice treated with carboplatin alone (Figure 7A). Therefore, the addition of CB[7] to carboplatin can lead to both an increase in acute toxicity and an increase in the antitumor effect of the drug, which requires further study when using lower doses.

The complex of oxaliplatin with CB7 did not demonstrate acute toxicity during the treatment of mice. Significant differences between the tumor size in the group of mice receiving injections of oxaliplatin and the group with the CB[7]–oxaliplatin treatment were not observed (Figure 7B). The comparable effect of oxaliplatin with CB[7] and free oxaliplatin is consistent with published data, which demonstrated that the complex of platinum preparations with CB[7] had a similar antitumor effect compared to the free drug [15,17].

**Figure 8:**
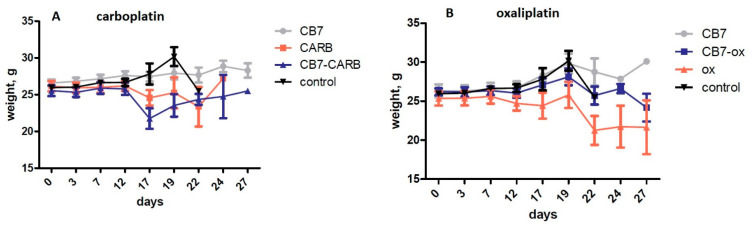
Effects of the CB[7] and platinum drugs on mice body weight in melanoma mice model. (**A**) Impact of the mixture of CB7 and carboplatin (1:1) on mice weight. (**B**) Impact of the oxaliplatin–CB[7] complex on mice weight.

Platinum drugs are known to have many side effects, including weight loss. It was shown that the CB[7]–oxaliplatin complex caused significantly lower weight loss in mice compared to free oxaliplatin on day 4 after treatment (Figure 8B), which indicates a decrease in side effects. It is known that oxaliplatin-treated mice displayed reduced weight gain, which was associated with myotoxicity [26]. Additionally, oxaliplatin-treated mice had a significant decrease in body weight compared to the naïve mice [27]. According to published data, a similar effect was already observed in studies of antitumor properties of the CB[7]–oxaliplatin complex in the HCT116 xenograft tumor model [28]. The higher safety is probably caused by the host–guest interaction between oxaliplatin and CB[7]. For carboplatin with CB[7], on the contrary, there was a tendency to decrease the mouse’s body weight compared to carboplatin on the day after the administration of drugs (Figure 8A); however, later the difference was leveled. Perhaps this effect is associated with increased toxicity of CB[7] with carboplatin.

Thus, the presence of CB[7] has different effects on different platinum drugs. For carboplatin, addition of CB[7] increased the antitumor effect, but also increased the toxicity of the drug. For oxaliplatin, CB[7] did not enhance the antitumor effect of the drug, but at the same time, it can reduce the side effects of oxaliplatin.

## 3. Materials and Methods

### 3.1. Materials

CB[7] was synthesized according to the standard protocol described in [29]. Carboplatin was purchased from Tokyo Chemical Industry Co., (Tokyo, Japan), and oxaliplatin was purchased from Biomol (Hamburg, Germany). RPMI-1640 medium and phosphate-buffered saline (PBS) and L-glutamine were obtained from Biolot (Saint Petersburg, Russia). HyClone fetal calf serum was obtained from GE Healthcare (Chicago, IL, USA).

The inclusion complex of CB[7] and oxaliplatin was synthesized according to the protocol in [17], with some modifications. Although in [18] it was suggested that the mixture of oxaliplatin and CB[7] should be heated at 100 °C for one day, we have found that such a long heating may lead to partial decomposition of the initial reagents. Therefore, we stopped the heating after 6 h and obtained the water solution of the oxaliplatin–CB[7] complex. The formation of the complex and the absence of impurities in the solution were confirmed with ^1^H NMR spectroscopy (Figure S3 in Supplementary). The diffusion-ordered 2D NMR spectra were also registered to confirm the formation of the complex (Figure S5 in Supplementary). The diffusion rate is ~2 × 10^−12^ which is much lower than the diffusion rate of free CB[7] [30], meaning that the complex is bigger and heavier than free CB[7]. All NMR experiments were performed on a Bruker Avance III 500 MHz spectrometer at room temperature (25 °C). Behavior and stability of the CB[7]–oxaliplatin complex in RPMI-1640 was thoroughly studied in [31].

### 3.2. Cell Cultures

Peripheral blood mononuclear cells (PBMCs) were isolated from blood samples using a standard Ficoll-Urografin density gradient method (d = 1.077 g/cm^3^) [32]. Blood samples were obtained from 21 healthy individuals (mean age: 36.0 ± 2.48 years) and 10 patients with B-cell lymphomas, including 6 patients with chronic lymphocytic leukemia and 4 patients with mantle cell lymphoma (mean age: 63.1 ± 2, 14 years). Informed written consent was obtained from all subjects. This study was approved by the Ethics Committee of RIFCI.

The chronic myelogenous leukemia K562 cell line was obtained from N.N. Blokhin NMRCO (Moscow, Russia). The murine melanoma cell line B16 was kindly provided by Dr. G.V. Seledtsova (Laboratory of Cellular Biotechnologies RIFCI, Novosibirsk, Russia). Cells were cultured in RPMI-1640 medium with 10% FBS in 25 cm^2^ flasks at 37 °C under 5% CO_2_ in air with high humidity.

Cells were cultured at 37 °C in a 5% CO_2_ humidified atmosphere for 2 days (B16 and K562 cells) or 3 days (PBMC). The cultures were performed in the presence of the CB[7]–oxaliplatin complex or carboplatin in mixture with CB[7] (1:1) in different concentrations. As a control, we used non-treated PBMCs, PBMCs cultured with oxaliplatin, carboplatin or CB[7].

### 3.3. Cell Viability Assay

The cytotoxic effect was evaluated in cultures after 48 (B16, K562) or 72 h (PBMC) using the MTT assay for adherent cell lines and WST-1 assay for suspension cultures. B16 and K562 cells (10^4^ cells/well) and PBMCs (10^5^ cells/well) were cultured in a 96-well plate (Costar, UK). As a positive control, 10% DMSO was used.

### 3.4. Cell Proliferation Assay

B16 and K562 cells were seeded at 1.5 × 10^5^ cells/well into a flat-bottomed 24-well plate (Costar, UK). PBMCs (1 × 10^6^/mL) were cultured using 48 flat-bottom culture plates (Costar, UK); anti-CD3 antibodies (1 μg/mL) and recombinant human IL-2 (100 units/mL) were added for the PBMCs’ activation. To evaluate cell proliferation, cells were labeled with 5,6-carboxyfluorescein diacetate succinimidyl ester (CFSE) (0.004 mM) (Invitrogen, Eugene, OR, USA) before cultivating. Then, cells were harvested and washed in 1 mL of phosphate-buffered saline containing 0.5% FBS. In addition, to evaluate the proliferation of lymphocyte subsets, PBMCs were stained with monoclonal anti-human antibodies (CD45-PE/Cy7, CD3-APC, CD4-PerCP/Cy5.5, CD19-PE, all from BioLegend, (San Diego, CA, USA)). Analyses were performed using a FACSCantoII (Becton Dickinson, NJ, USA) and FACSDiva software (Becton Dickinson, NJ, USA).

### 3.5. In Vivo Studies

Antitumor effects of oxaliplatin and carboplatin with CB[7] in vivo were evaluated in a murine B16 cell line-based melanoma model. Wild-type female C57BL/6 mice were obtained from Goldberg Research Institute of Pharmacology and Regenerative Medicine (Tomsk, Russia). The experiments were conducted according to the institutional ethical guidelines for animal experiments. The B16 cells were syngeneic with the C57BL/6 mice used for the vaccination. B16 cells were subcutaneously injected under the scruff of C57BL/6 mice (10^5^/mice) to allow tumor growth. Animals were randomly divided into 7 groups with 8-10 mice in each group, and treatment was started twelve days later. Mice were treated with a single intraperitoneal injection of PBS (group 1); 3 mg/kg of oxaliplatin in PBS (group 2); CB[7] in PBS in equivalent molar concentration to oxaliplatin (group 3); oxaliplatin-CB7 complex in equivalent molar concentration to oxaliplatin (group 4); 120 mg/kg of carboplatin in PBS (group 5); CB[7] in PBS in equimolar quantity of carboplatin (group 6), and CB7 with carboplatin (group 7). Mice were euthanized when the tumor size reached >15 mm in mean diameter.

### 3.6. Statistical Analysis

All experimental data were expressed as means ± standard error of the mean (SEM) or median (25th-75th percentile). Differences between groups were evaluated for statistical significance using a Student’s *t*-test or Mann–Whitney test when the data were not normally distributed. A *p*-value < 0.05 was regarded as the minimum criteria for statistical significance. For in vivo studies, the Kaplan–Meier method and log-rank test using GraphPad Prism software package version 5.0 (GraphPad Software, Inc., San Diego, CA, USA) were used to compare the survival rates between groups. Differences were considered significant if *p* < 0.05.

## 4. Conclusions

We studied the biological properties of the CB[7]–oxaliplatin inclusion complex and carboplatin in mixture with CB[7] at a 1:1 ratio. In both cases, CB[7] did not enhance the cytotoxic effect of the platinum(II) drugs on the primary cell culture (peripheral blood mononuclear cells) in vitro. The CB[7]–oxaliplatin complex had a greater cytotoxic effect on the tumor cell lines B16 and K562 in vitro, compared to free oxaliplatin. However, in vivo studies did not show a significant difference between the CB[7]–oxaliplatin complex and free oxaliplatin antitumor effects. At the same time, complexation with CB[7] reduced the acute toxicity of the oxaliplatin in vivo.

Despite the fact that carboplatin does not form a stable inclusion complex with CB[7], we found that the addition of this nanosized cavitand affects the biological properties of carboplatin (Table 3). This fact could be related to the complexation of carboplatin metabolites with CB[7] and an enhancement of their biological properties, to the previously observed ability of CB[7] to induce carboplatin aquation [20] or, probably, to the encapsulation of some medium components that may affect the carboplatin’s biological activity. Thus, the mixture of carboplatin with CB[7] has a more pronounced antitumor effect on the murine B16 melanoma cell line than free carboplatin. However, the tumor cells with a bone marrow precursor origin (K562) may be more viable when cultivated with carboplatin in mixture with CB[7]. In addition, CB[7] also significantly increased the acute toxicity of the carboplatin in vivo.

## Figures and Tables

**Table 1:** The relative number and level of spontaneous proliferation of various subpopulations of PBMCs.

	CD3^+^CD4^+^		CD3^+^CD4^−^		CD19^+^	
	Frequency (%)	Dividing Cells (%)	Frequency (%)	Dividing Cells (%)	Frequency (%)	Dividing Cells (%)
control	49.8 (47.3–59.0)	1.8 (0.9–2.4)	25.3 (19.1–31.7)	3.1 (0.9–3.9)	7.0 (3.5–7.6)	2.4 (1.2–3.4)
CB[7] 0.3 M	53.7 (45.3–62.7)	2.3 (1.0–2.4)	26.6 (20.0–32.0)	3.3 (1.1–4.1)	4.7 (2.7–6.3)	2.7 (0.9–4.0)
carboplatin 0.3 M	55.3 (45.3–58.7)	2.2 (0.9–4.3)	26.4 (20.8–31.4)	4.5 (1.2–9.6)	4.8 (1.9–10.6)	2.7 (1.0–3.4)
carboplatin:CB[7] 1:1 0.3M	60.3(52.8–66.6) *#	2.5 (1.4–3.7)	27.6 (22.1–33.1)	4.3 (1.6–4.7)	5.9 (1.7–7.8)	3.1 (0.6–3.5)
CB[7] 0.1 M	52.7 (42.9–55.5)	2.4 (1.2–3.0)	27.4 (25.7–31.2)	4.0 (1.1–7.0)	5.6 (2.6–6.5)	2.8 (1.9–3.5)
oxaliplatin (0.1 M)	71.6 (63.5–76.6) *	2.5 (1.1–3.9)	21.4 (21.1–22.9) *	4.6 (1.9–8.8)	1.2 (0.9–4.1) *	1.4 (0.6–2.1) *
CB[7]–oxaliplatin 0.1 M	73.4 (69.1–77.2) *	2.4 (1.1–4.1)	21.6 (17,9–23.2) *	5.1 (1.6–6.6)	1.8 (1.2–2.8) *	2.0 (1.0–2.5)

* Indicates a significant difference (*p* < 0.05) vs. control; # Indicates a significant difference (*p* < 0.05) vs. carboplatin.

**Table 2:** The relative number and level of anti-CD3 stimulated proliferation of various subpopulations of PBMCs.

	CD3^+^CD4^+^		CD3^+^CD4^−^		CD19^+^	
	Frequency (%)	Dividing Cells (%)	Frequency (%)	Dividing Cells (%)	Frequency (%)	Dividing Cells (%)
control	51.3 (41.2–59.2)	28.7 (19.0–37.3)	29.9 (23.8–45.4)	30.6 (27.2–32.6)	5.4 (2.0–6.2)	5.3 (4.1–14.9)
CB[7] 0.3 M	51.5 (41.1–59.9)	27.4 (12.6–29.8)	30.2 (23.2–50.5)	28.9 (22.8–30.1)	4.2 (1.7–5.9)	5.3 (3.2–9.6)
carboplatin 0.3 M	48.7 (36.8–50.8)	2.7 (2.4–5.1) *	24.3 (20.2–27.9)	4.2 (3.6–7.8) *	4.9 (1.9–10.1)	2.8 (1.1–4.0) *
carboplatin:CB[7] 1:1 0.3M	50.4 (40.5–65.8) #	2.5 (1.5–4.8) *	22.1 (19.4–28.6) *	4.8 (3.1–6.7) *	3.1 (1.5–9.1)	1.7 (1.1–2.5) *
CB[7] 0.1 M	46.3 (41.9–55.5)	21.1 (17.1–30.1)	32.0 (29.6–45.0)	30.0 (27.4–30.6)	4.0 (2.3–6.9)	4.9 (4.0–6.4)
oxaliplatin 0.1 M	63.8 (60.9–69.7) *	2.8 (1.4–3.9) *	19.8 (16.4–21.4) *	5.5 (2.4–9.4) *	1.5 (1.1–3.7) *	2.8 (1.4–3.7)
CB[7]–oxaliplatin 0.1 M	65.0 (62.4–71.6) *	2.8 (1.6–4.6) *	19.9 (17.8–27.5) *	5.7 (2.4–7.2) *	1.9 (0.9–2.2) *	3.6 (2.7–4.3) †

* Indicates a significant difference (*p* < 0.05) vs. control; # Indicates a significant difference (*p* < 0.05) vs. carboplatin; † Indicates a significant difference (*p* < 0.05) vs. oxaliplatin.

**Table 3:** Effects of CB[7] on the biological properties of platinum drugs.

	Carboplatin	Oxaliplatin
Increased cytotoxicity for tumor cell lines	+	++
Deceased cytotoxicity for non-tumor cells	0	0
Decreased immunosuppression in vitro	0	0
Increased antitumor activity in vivo	+	0
Decreased side effects in vivo	−	+

++ (very beneficial, strong effects), + (moderate effects), 0 (no influence), − (not valuable, impairment of biological effects).

## Supplementary Materials

The following are available online at https://www.mdpi.com/article/10.3390/ijms22147337/s1.
