# Supplementary material for: The Effect of Cucurbit[7]uril on the Antitumor and Immunomodulating Properties of Oxaliplatin and Carboplatin
Source: Int J Mol Sci. 2021 Jul 8;22(14):7337. doi: 10.3390/ijms22147337 (PMC8303694; doi:10.3390/ijms22147337)
Supplement: Supplementary file 1 [file ijms-22-07337-s001.zip › ijms-1262320-supplementary.pdf]

Supplementary

Table of Contents

Figure S1. Molecular structures of CB[7], oxaliplatin and carboplatin. Atomic coordinates taken from ref. [19]. **p.2**

Figure S2. The structure of the inclusion complex oxaliplatin@CB[7]. Atomic coordinates taken from ref. [19]. **p.2**

Figure S3. ^{1}H NMR spectra of CB[7] (green), oxaliplatin (red) and the inclusion complex oxaliplatin@CB[7] (blue) in D_2O. **p.3**

Figure S4. ^{1}H NMR spectra of the inclusion complex oxaliplatin@CB[7] in H_2O with solvent suppression (zgpr). **p.3**

Figure S5. ^{1}H diffusion ordered (DOSY) NMR spectrum of the inclusion complex oxaliplatin@CB[7] (blue) in D_2O. **p.4**

Figure S6. ^{1}H NMR spectrum of the mixture of carboplatin and CB[7] **p.5**

Figure S1. Molecular structures of CB[7], oxaliplatin and carboplatin. Atomic coordinates taken from ref. [19].

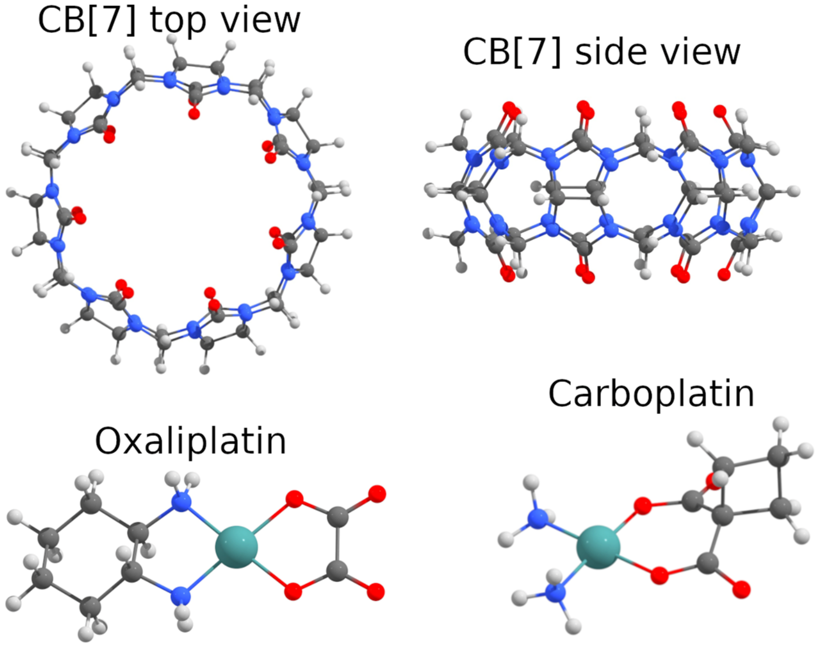

Figure S2. The structure of the inclusion complex oxaliplatin@CB[7]. Atomic coordinates taken from ref. [19].

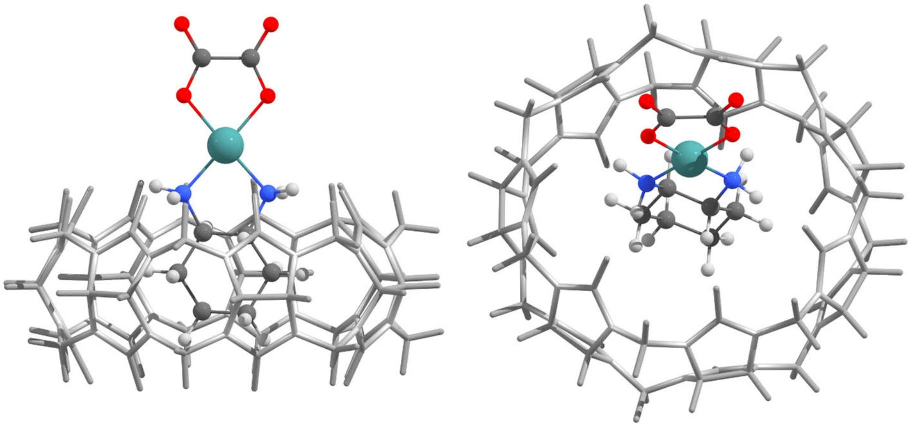

Figure S3. ^{1}H NMR spectra of CB[7] (green), oxaliplatin (red) and the inclusion complex oxaliplatin@CB[7] (blue) in D_2O.

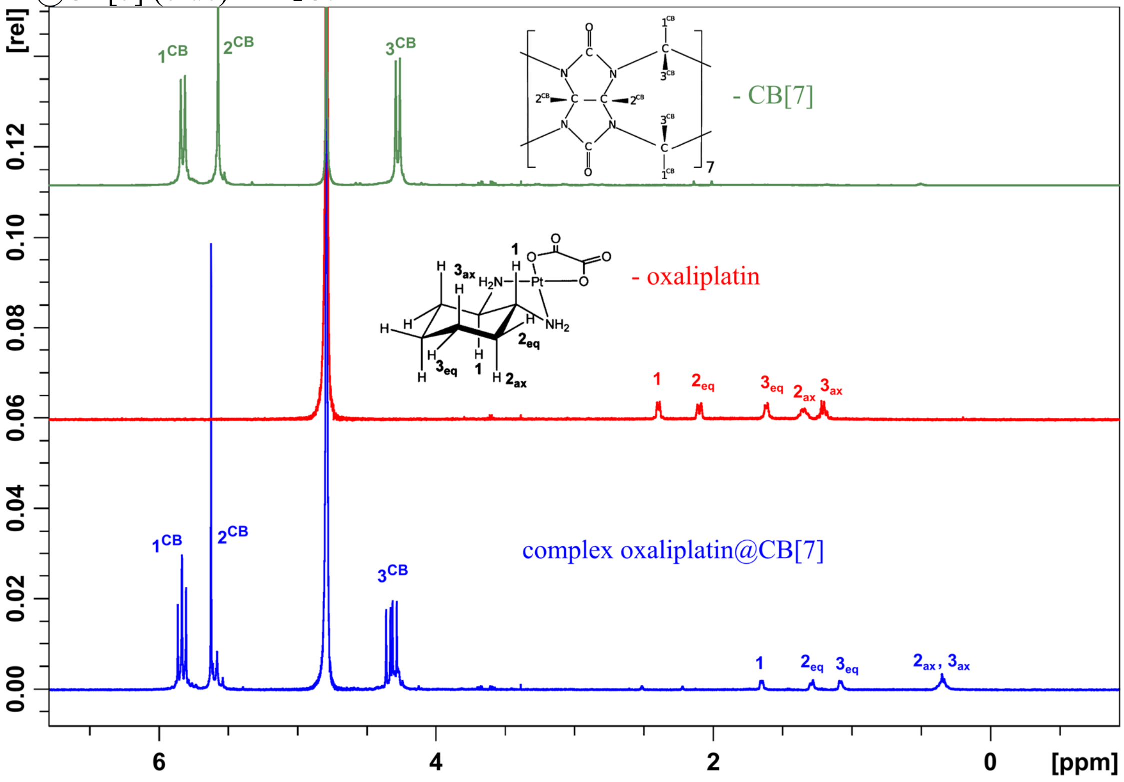

The peak positions agree well with the data in ref.[17].

Figure S4. ^{1}H NMR spectra of the inclusion complex oxaliplatin@CB[7] in H_2O with solvent suppression (zgpr).

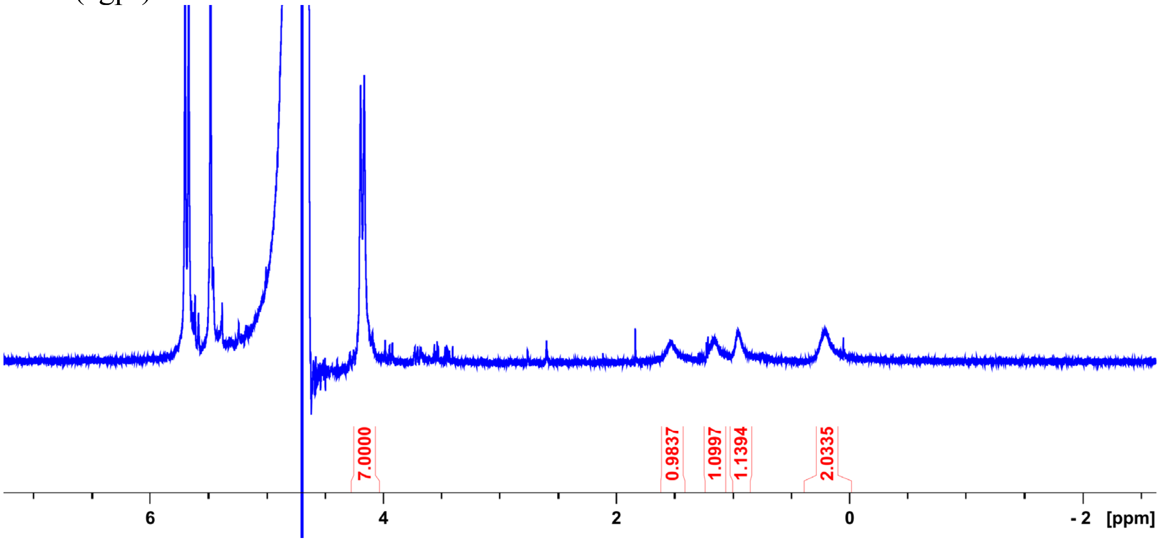

In water the spectrum looks similar. The ratios between peak integrals indicate that CB[7]:oxaliplatin is 1:1. No significant excess of CB[7] or oxaliplatin.

Figure S5. ^{1}H diffusion ordered (DOSY) NMR spectrum of the inclusion complex oxaliplatin@CB[7] (blue) in D_2O.

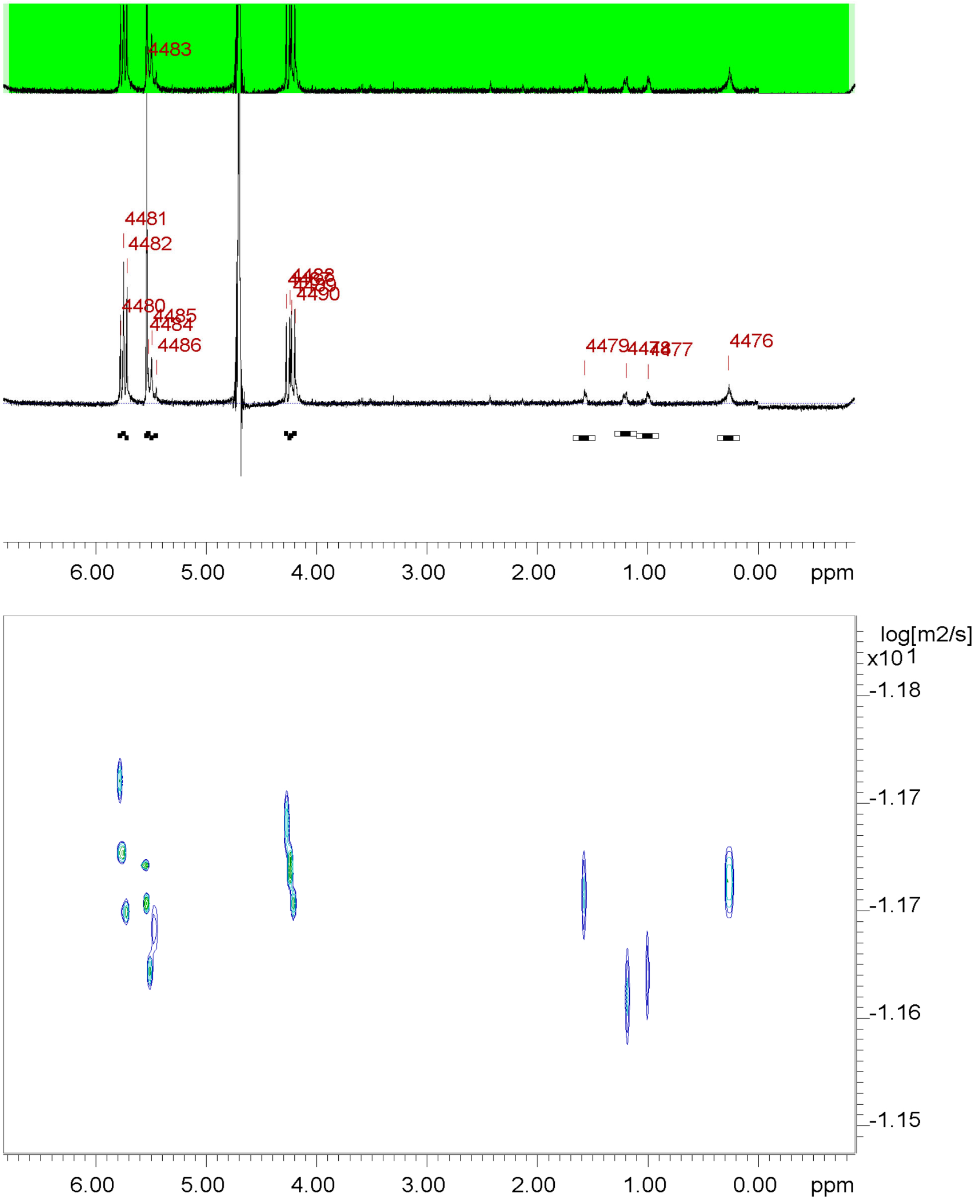

The method shows that the complex moves as one. **The diffusion rate is ~$2*10^{-12}$** which is much lower than the diffusion rate of free CB[7] [27] meaning that the complex is bigger and heavier than free CB[7].

Figure S6. ^{1}H NMR spectrum of the mixture of carboplatin and CB[7]

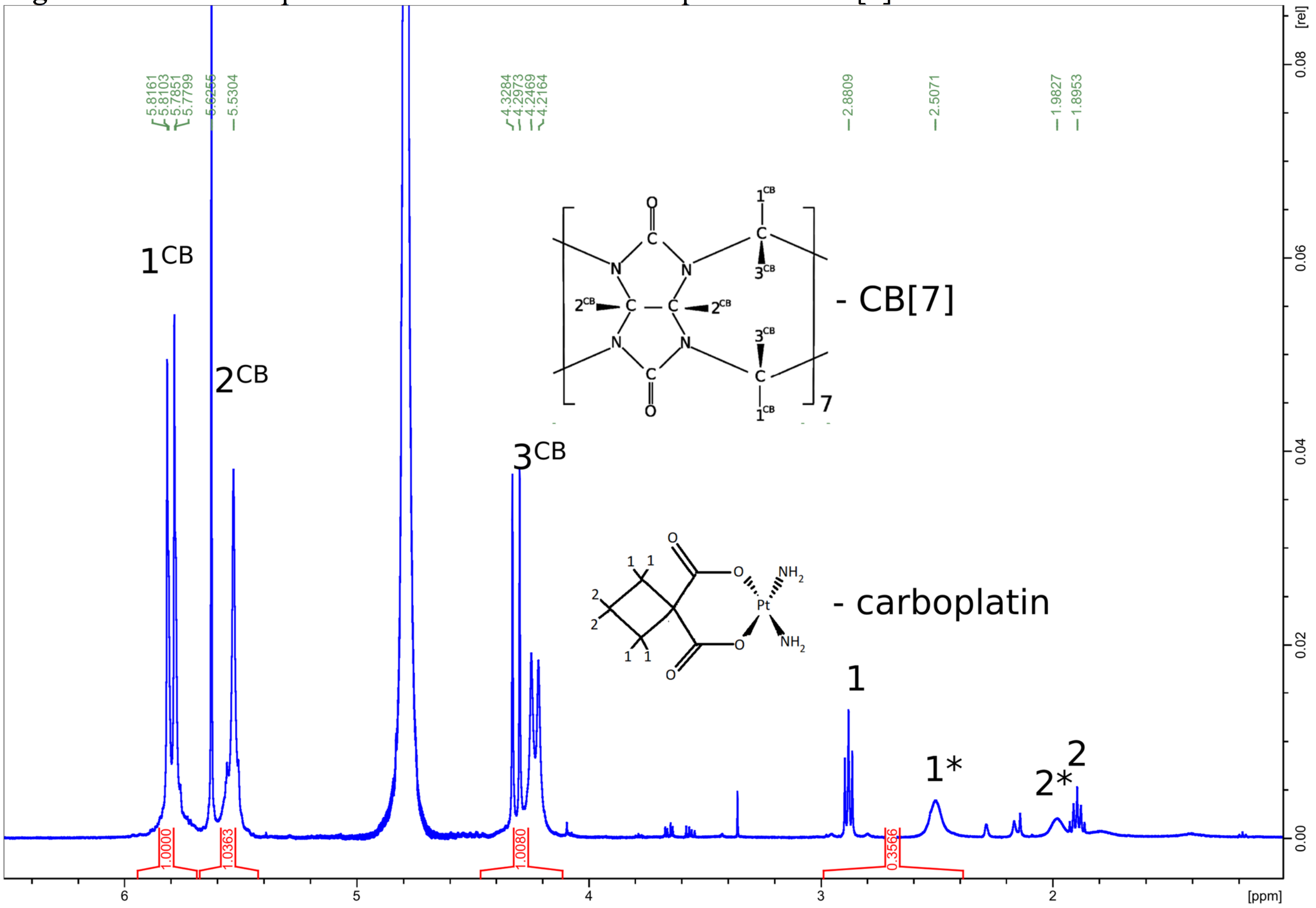

More information on the behavior of the mixture of carboplatin and CB[7] may be found in references [20] and [22].
